# Role of post-transplant graft scintigraphy in kidney donation after circulatory death

**DOI:** 10.3389/frtra.2022.1065415

**Published:** 2022-12-19

**Authors:** Manon Belhoste, Gilles Allenbach, Thomas Agius, Raphael P. H. Meier, Jean-Pierre Venetz, Jean-Marc Corpataux, Antoine Schneider, Déla Golshayan, John O. Prior, Sébastien Déglise, Marie Nicod-Lalonde, Alban Longchamp

**Affiliations:** ^1^ Department of Vascular Surgery, Lausanne University Hospital, Lausanne, Switzerland; ^2^ Department of Nuclear Medicine and Molecular Imaging, Lausanne University Hospital, Lausanne, Switzerland; ^3^ Department of Biomedical Sciences, University of Lausanne, Lausanne, Switzerland; ^4^ Department of Surgery, University of Maryland School of Medicine, Baltimore, MD, United States; ^5^ Transplantation Center, Lausanne University Hospital, Lausanne, Switzerland; ^6^ Adult Intensive Care Unit, Lausanne University Hospital, Lausanne, Switzerland

**Keywords:** kidney transplantation, scintigraphy, outcome, DCD−donation after cardiac death, tranplantation

## Abstract

**Background:**

There is no consensus on how to predict post-transplant function of donation after circulatory death (*DCD*) kidneys. Thus, we aimed to identify renal scintigraphy parameters that could predict 1-year kidney function.

**Methods:**

In this single center study, we included all consecutive DCD kidney recipients between 2013 and 2021 (*n* = 29). Patients who did not have a scintigraphy within 10 days of transplantation (*n* = 3), recipients of multiple organs and less than 18 years old were excluded (*n* = 1). Primary endpoint was the estimated glomerular filtration rate (eGFR).

**Results:**

Median eGFR and serum creatinine at 1 year were 67 µmol/L (56–81) and 111 ml/min (99–132), respectively. Among parameters tested, the 3rd/2nd-minute activity ratio had the best diagnostic performance (AUC: 0.74 and 0.71, for eGFR and creatinine) 1 year post transplantation. Using 1.21 as the best cut off, the 3rd/2nd-minute activity ratio specificity and sensitivity to predict eGFR >60 ml/min was 0.82 and 0.83. Renal function was significantly better at 1 week, 3, 6, and 12 months after transplantation in patients with 3rd/2nd-minute activity ratios above 1.21.

**Conclusion:**

This study suggests that the 3rd/2nd-minute activity ratio can predict graft function at 1 year. The benefit of post-transplant scintigraphy should be further validated in a prospective cohort.

## Introduction

Organ transplants are meeting less than 10% of global demands. In the United States, 13 people die each day while waiting for a kidney transplant (1). This shortage has led to an interest in expanding the donor pool with donors after circulatory death (DCD) (2, 3). Compared with standard-criteria grafts, the use of DCD kidneys is associated with an increased rate of delayed graft function (DGF) (4), higher rates of biopsy-proven acute rejection, and higher incidence of overall graft failure (5). Besides a higher risk of local immune activation and subsequent rejection (6), the presence of DGF is associated with an increased length of hospital stay, increasing patient's morbidity and transplantation-related costs (7). However, the relationship between DGF, or other early surrogates of kidney graft function, and long-term graft and patient survival remains uncertain. In addition, the reported incidence of DGF greatly varies from 18% to 55% (8, 9). Such variability results from ambiguity in the definition of the event, differences in reporting, as well as inconsistency in donors characteristics that are analyzed (9, 10). While persistent inflammation in protocol biopsies performed within the first 6 months post-transplantation correlates with decreased renal function at 1 and 2 years (11), the procedure may be associated with relevant complications (e.g., hemorrhage or arteriovenous fistula) (12). In this context, research has focused on identifying urinary and blood biomarkers, and non-invasive imaging modalities capable of predicting renal function (13).

Renal scintigraphy (RS) has a wide range of clinical applications such as the assessment of suspected obstructive nephropathy (14) and the evaluation of renal and urinary tract malformations (15). In the context of transplantation, RS accurately assesses the occurrence of tubular necrosis, as well as vascular and post-renal complications (16). Renogram grades from 0 to 5 were shown to correlate with 1- and 5-year graft survival in recipients of living and donated after brainstem death (DBD) kidneys (17). Despite the ability to predict survival of DBD kidney transplants in some studies, there is no consensus on the systematic use of RS after transplantation, the timing, and which parameters to rely on to predict kidney function.

Accurate post-operative estimation of graft function and survival is important since it impacts hospital stay, patient anxiety, the number of necessary tests and use of dialysis facilities. We recently analyzed the ability of standard and novel RS parameters to predict kidney function recovery in a cohort of patients with acute renal failure. In this setting, the most accurate parameters to predict recovery were the 3rd/2nd-minute activity ratio*,* and the 2nd/3rd-minute slope. Thus, in the present study we examined the ability of these newly developed RS parameters to predict kidney function and graft survival following DCD kidney transplantation.

## Materials and methods

### Study population, setting, and data collection

All consecutive patients who underwent renal transplantation from donation after circulatory death, between January 2013 and December 2021 in our institution (Lausanne University Hospital) were retrospectively analyzed. Those who did not undergo renal scintigraphy (RS) within 10 days post-transplantation (*n* = 3) or underwent repeat kidney transplantation (*n* = 1) or were <18 years old (*n* = 0) were excluded. Hence, a total of 29 patients (16 males, 13 females) were included in this study. The study was conducted in accordance with the Declaration of Helsinki and approved by the local ethics committee (CER-VD #2021–00081). Demographics and clinical data were collected from each patient's electronic medical records. Donor characteristics were retrieved from donor information sheets, and included age, last serum creatinine level, cold and warm ischemia time. Warm ischemia was defined as the time of agonal phase onset to the time when core cooling is initiated. Vascular anastomosis time was the time between the end of the cooling period to successful renal artery anastomosis and perfusion of the donor kidney. All laboratory tests and radiologic assessments (including RS) were performed at the discretion of the treating physician.

### Outcome measures

Data were analyzed to determine the correlation between early post-operative RS and kidney function (primary outcome). Kidney function was assessed by serum creatinine levels (sCr) and estimated glomerular filtration rate (eGFR) using the Cockcroft-Gault equation at 1 week, 3-, 6-, and 12-months post-transplantation.

### Dynamic renal scintigraphy

A 20 min dynamic acquisition using a 128 × 128 matrix (30 × 1s frame followed by 117 × 10s frame) was performed after injection of 20–50 MBq of Tc99m-MAG3 (*n* = 15) or I123-HIPPURAN (*n* = 14) on a single head gamma-camera (Millennium MPR, General Electrics, Waukesha, WI). To calculate quantitative renal uptake, we measured a kidney phantom filled with a standardized activity after each scan. A correction for attenuation was systematically applied. The first 30 s were reconstructed in 1-s and 3-s images for optimal visual evaluation of the arterial phase. A delay between the vascular activity (defined as the time between blood arrival in the iliac artery and the renal graft) was reported when >3 s. In addition, visual grading of time activity curves was done as previously published (16). A rising curve (Grade 3–4) was considered due to acute tubular necrosis. The following parameters were considered and collected: cortical perfusion index (derived from the ratio of areas under the arterial and renal curves), time to maximal vascular uptake (vascular peak time), time to maximal renal uptake (renal graft peak time), the median bladder appearance time (time between injection and the appearance of radioactive urine in the bladder), tracer's elimination half-life, accumulation index (percentage of total injected activity excreted by the kidney within one minute between 30 to 50 s and 90 to 110 s post-injection), elimination index [ratio of renal activity at peak time (max 3 min) to the renal activity at 20 min], accumulation slope (slope of the TAC during the one minute interval ). Serial one-minute renal absolute activity counts of the graft during the 2nd and 3rd minutes post-injection were extracted, and additional parameters were computed for the transplanted kidneys: renal activity ratio and activity slope between the 2nd and 3rd minutes (3rd/2nd-minute activity ratio, and 2nd/3rd-minute slope, respectively).

### Study definitions

Coexisting conditions were ascertained from physician documentation. DGF was defined as the need for at least one hemodialysis treatment in the first week after kidney transplantation or a failure of serum creatinine to fall by at least 10% on three consecutive days in the first week post-operatively (18).

### Statistical analysis

Descriptive statistics were used to report the data; results are reported as medians and interquartile ranges (IQR) or means and standard deviations, as appropriate. Categorical variables were summarized as numbers and percentages. A logistic regression analysis was used to analyze the association of single scintigraphy parameters, with kidney function. Sensitivity, specificity (with binomial 95% confidence intervals, CI), positive predictive value, negative predictive value (with bootstraps 95% confidence intervals) as well as the area under the Receiver operating characteristic (ROC) curve (AUC) were calculated using the (pROC) and (cutpointr) packages. The optimal cutpoint was set to maximize the sum of sensitivity and specificity. No imputation was made for missing data. Analysis were performed with R (RStudio v1.4.1717).

## Results

Twenty-nine patients fulfilled the inclusion criteria and were analyzed. Their baseline characteristics are shown in Table 1. Median recipient age was 53 (25–73) years and 45% were female. Median body mass index (BMI) prior to transplantation was 27.8 (21.6–43) kg/m^2^. The median duration on dialysis prior to transplantation was 56 months (0–102). Two (7%) patients underwent a preemptive transplantation. Median donor age was 53 (14–65) years. Median pre-transplant serum creatinine levels (sCr) in donors was 71 (32–147) µmol/L.

**Table 1:** Baseline patient characteristics.

Characteristic and Clinical data	Patients *(N = 29)*
Recipient characteristics	
Age, year – (range)	53 (25–73)
Sex – *n* (%)	
Male	16 (55)
Female	13 (45)
Coexisting disorders – *n* (%)	
Hypertension	23 (79)
Cardiovascular disease^a^	12 (41)
Diabetes mellitus	8 (28)
Body mass index, kg/m^2^ – Median (IQR)	27.8 (21.6–43)
Pre-emptive transplantation – *n* (%)	2 (7)
Pre-transplant dialysis duration, months – Median (IQR)	56 (0–102)
Donor characteristics	
Age, yr – (range)	52 (14–65)
Warm ischemia time, min – Median (IQR)	33 (25–124)
Cold ischemia time, min – Median (IQR)	459 (217–886)
Last serum creatinine, µmol/L – Median (IQR)	71 (32–147)
Transplantation characteristics	
Anastomosis time, min – Median (IQR)	30 (13–69)
Allograft with >1 renal artery – *n* (%)	6 (21)
Renal function post transplantation	
Day of the renal scintigraphy	2 (1–10)
Delayed graft function – *n* (%)	18 (62)
Length of hospital stay, days – Median (IQR)	22 (13–79)
Serum creatinine at 1 week, µmol/L – Median (IQR)	475 (301–608)
Serum creatinine at 3 months^b^, µmol/L – Median (IQR)	122 (104–137)
Serum creatinine at 6 months^a^, µmol/L – Median (IQR)	113 (102–139)
Serum creatinine at 12 months^a^, µmol/L – Median (IQR)	111 (99–131)
eGFR at 1 week, ml/min – Median (IQR)	15 (12–24)
eGFR at 3 months^b^, ml/min – Median (IQR)	65 (52–77)
eGFR at 6 months^a^, ml/min – Median (IQR)	66 (51–79)
eGFR at 12 months^a^, ml/min – Median (IQR)	67 (55–81)
Biopsy-proven acute rejection – *n* (%)	1 (3)

^a^ Data were available for 27 of the 29 patients.

^b^ Data were available for 28 of the 29 patients.

Median warm, cold ischemia and vascular anastomosis time were 33 (25–124), 459 (217–886) and 30 (13–69) minutes, respectively. Median follow-up duration was 27 months (3–96). DGF was observed in 18 (62%) patients. One patient (3%) experienced a biopsy-proven acute rejection. During the first year, one graft (3%) was explanted due to early recurrence of complement-mediated thrombotic microangiopathy. Median sCr and eGFR at 1 year were 111 (99–131) µmol/L and 67 (55–81) ml/min, respectively.

**Figure 1:**
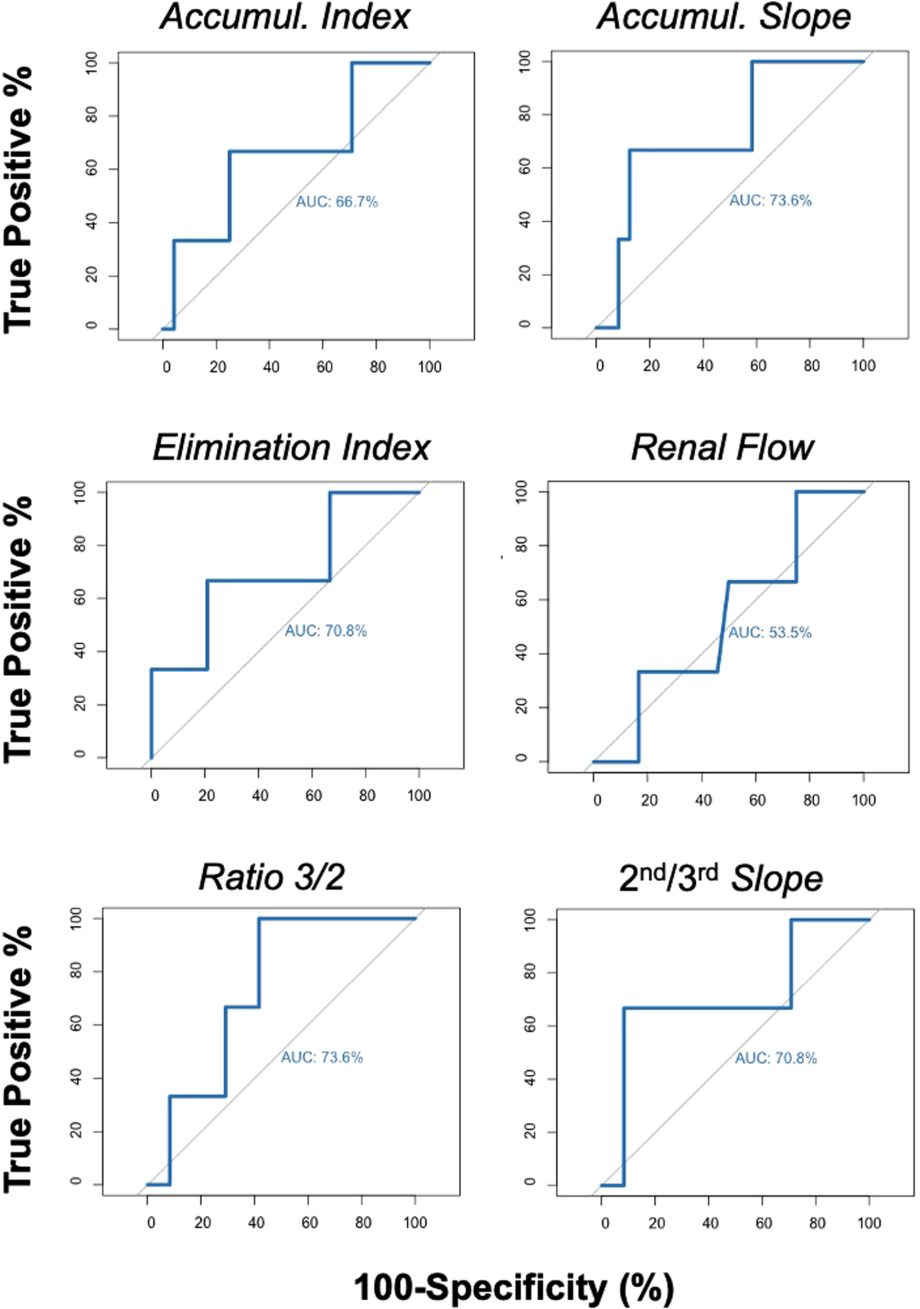
ROC curves illustrating the diagnostic ability of RS parameters to discriminate patients with serum creatinine levels above or below 80 µmol/L at 1 year.

Dynamic RS was performed in all patients between day 1–10 (median: 2) post transplantation. Table 2 provides quantitative and qualitative scintigraphy data. First, we assessed the diagnostic ability of various RS parameters to discriminate between patients with sCr levels at 1 year above and below 80 µmol/L using ROC curves. As shown in Figure 1, the AUC of the accumulation index, accumulation slope, elimination index, renal flow index, 3rd/2nd-minute activity ratio*,* and 2nd/3rd-minute slope were: 0.67 (95% CI, 0.27–1.00), 0.74 (95% CI, 0.41–1.00), 0.71 (95% CI, 0.31–1.00), 0.54 (95% CI, 0.18–0.89), 0.74 (95% CI, 0.50–0.97), and 0.71 (95% CI, 0.29–1.00), respectively. Of note, renal grading scale, cortical perfusion index, vascular and renal peak time, median bladder appearance time, and tracer elimination half-time did not discriminate between high and low sCr or eGFR levels at 1 year (AUC ≤0.5, data not shown).

**Table 2:** Quantitative and qualitative post-transplant scintigraphy data.

Quantitative and qualitative scintigraphy data	Patients *(N = 29)*
Accumulation index – Median (IQR)	4.18 (3.65–4.94)
Accumulation slope – Median (IQR)	0.55 (0.38–0.71)
Elimination index – Median (IQR)	0.55 (0.49–0.69)
Renal flow index – Median (IQR)	0.68 (0.20–1.18)
3rd/2nd-minute activity ratio – Median (IQR)	1.21 (1.17–1.24)
2nd/3rd Slope counts – Median (IQR)	1658.0 (1259.0–2131.0)

**Figure 2:**
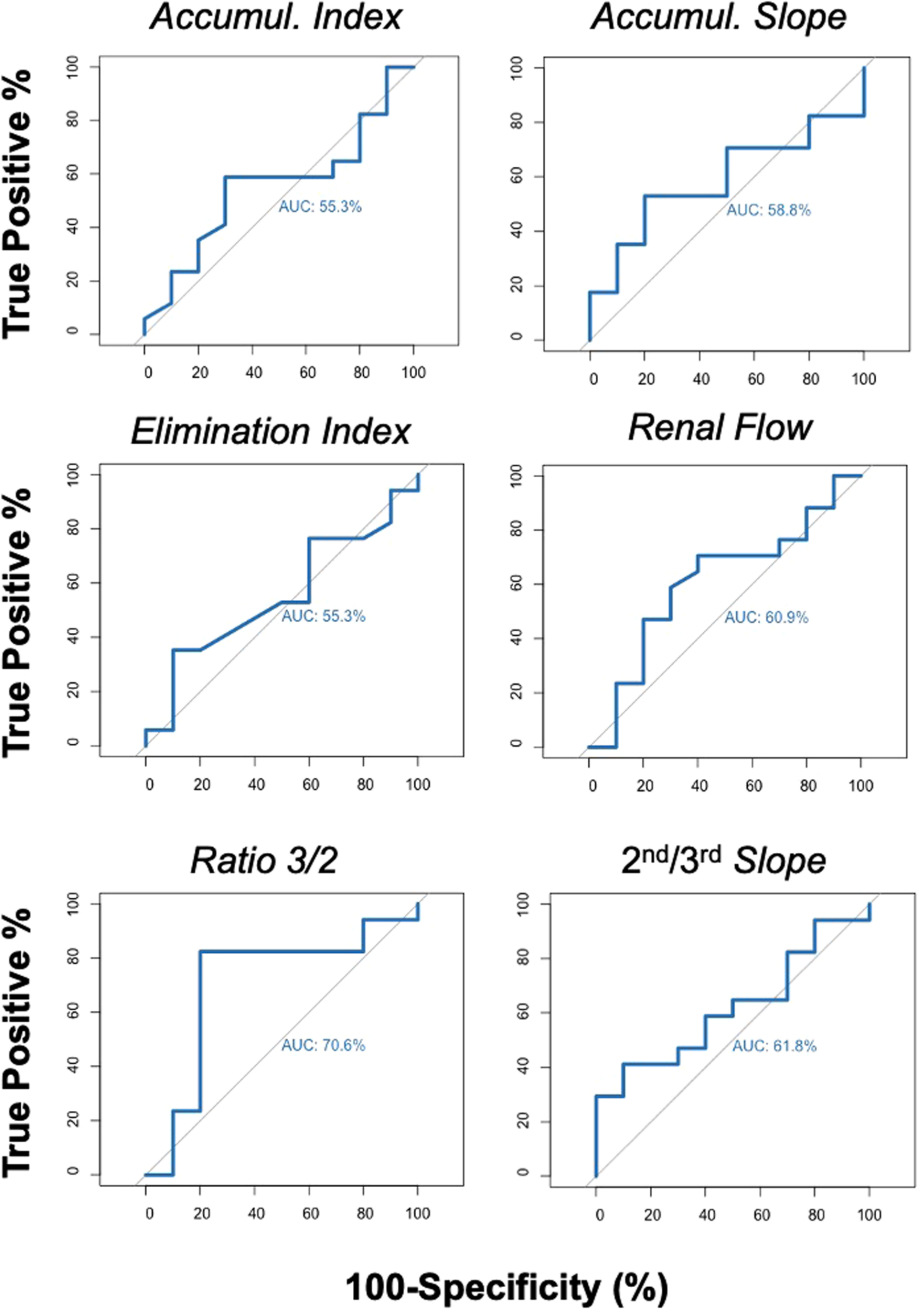
ROC curves illustrating the diagnostic ability of RS parameters to discriminate patients with eGFR above or below 60 ml/min at 1 year.

We similarly tested the ability of RS parameters to predicted eGFR above or below 60 ml/min at one year. The AUC of the accumulation index, accumulation slope, elimination index, renal flow index, 3rd/2nd-minute activity ratio*,* and 2nd/3rd-minute slope were: 0.55 (95% CI, 0.32–0.78), 0.59 (95% CI, 0.37–0.81), 0.55 (95% CI, 0.37–0.81), 0.61 (95% CI, 0.37–0.84), 0.71 (95% CI, 0.47–0.94), and 0.62 (95% CI, 0.40–0.86), respectively (Figure 2). Altogether, the 3rd/2nd-minute activity ratio appeared to have the strongest differentiating capacity between the two groups (high vs. sCr and eGFR at 1 year, Figures 1, 2).

Based on these findings, we established a RS score in attempt to improve the prediction of renal function at 1 year. The score combined the accumulation slope, slope counts and 3rd/2nd-minute activity ratio. A pre-established scale (1: low, 2: moderate, 3: high risk, 4: very high) was given to each RS parameter (total score 3–12, Supplementary Figure S1A). Using logistic regression and random forest model, the AUC to discriminate patients with sCr levels above and below 80 µmol/L were 0.79 and 0.74, respectively (Supplementary Figure S1B). When using eGFR above and below 60 ml/min, the AUC were 0.65 and 0.47, respectively (Supplementary Figure S1B). Thus, the score did not provide a better prediction model than the 3rd/2nd-minute activity ratio alone, which was considered for further analysis.

We then calculated the optimal cut-off value for the 3rd/2nd-minute activity ratio parameter, defined as the maximal sum of sensitivity and specificity (absolute maximum = 2). For sCr levels (80 µmol/L) and eGFR (60 ml/min), the best cut-off was 1.22 (1.22–1.24) and 1.21 (1.21–1.21), respectively (Table 3). Sensitivity and specificity were 1.0 (1.0–1.0) and 0.64 (0.55–0.75) for sCr, 0.83 (0.76–0.89) and 0.82 (0.71–0.90) for eGFR at 1 year.

**Table 3:** Diagnostic performance of the 3rd/2nd-minute activity ratio.

3rd/2nd minute activity ratio median (IQR)	Area under curve	Best cut-off	Sensitivity	Specificity
Serum creatinine level at 1 years^a^	0.74 (0.66–0.82)	1.22 (1.22–1.24)	1.0 (1.0–1.0)	0.64 (0.55–0.75)
eGFR at 1 year^a^	0.71 (0.64–0.80)	1.21 (1.21–1-21)	0.83 (0.76–0.89)	0.82 (0.71–0.90)

^a^ Data were available for 27 of the 29 patients.

**Figure 3:**
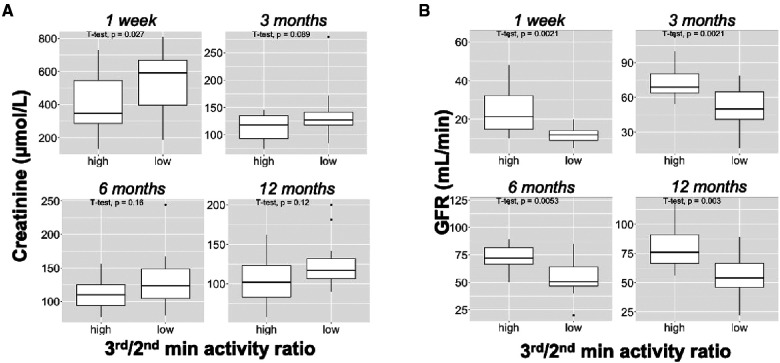
Identification of the optimal cut-off value to predict renal function. (**A,B**) Serum creatinine levels (**A**) and eGFR (**B**) at the indicated time in patients with a high (> or =1.21) or low (<1.21).

Interestingly, renal function was significantly higher at 1 week, 3 months, 6 months and 1 year in patients with a 3rd/2nd-minute activity ratio above 1.21 (Figures 3A, B). On the other hand, the presence or absence of DGF did not correlate with kidney function beyond 1 week (Supplementary Figures S1A,B). In the presence or absence of DGF, median eGFR and sCr levels at 1 year were 60 ml/min/1·73 m^2^ (55–76) and 102 µmol/L (90–123) vs. 50 ml/min (46–63) and 117 µmol/L (104–132), respectively (Supplementary Figures S2A,B).

## Discussion

In this retrospective study including 29 DCD kidney transplant recipients, we found that early post-operative 3rd/2nd-minute activity ratio on RS alone could predict kidney function at one year. We found that this new index, calculated based on renal counts in the first 3 min had better prognostic abilities, than conventional RS parameters such as the accumulation index, accumulation slope, elimination index and renal flow index or even a complex score.

Beyond RS, several clinical scores such as donor risk score index (19), or scores that include cold ischemia time, recipient BMI, donor age, last donor sCr, and administration of anti-thymocyte globulin are used to predict kidney function (20). DGF is an outcome of interest in an era where renal allografts increasingly originate from marginal donors, such as extended criteria and DCD donors. In this cohort, the occurrence of DGF did not significantly affect graft function beyond 1 week post-operatively. While DGF is a well-described risk factor for long-term graft survival in DBD donor kidney transplantation (4, 21), in particular in the presence of acute rejection (6, 22, 23), this association is much less clear with controlled DCD donor kidney transplantation (24–26). Interestingly, this form of resilience of DCD kidneys following DGF was attributed to a downregulation of intra-graft pro-inflammatory pathways, as well as upregulation of cell proliferation suppressor pathways in a cohort comparing DBD and DCD kidney transplants (27). The lack of association between early DGF and long-term graft function in DCD donor kidney transplantation may also be because of the subjectivity of using DGF as an outcome measure, as well as differences in definitions and early post-transplant dialysis prescriptions between clinicians and centers (28, 29).

Kidney graft function in this cohort was similar to other studies, which was reported between 43 and 53 ml/min at 1 year for DCD grafts (30, 31). In our cohort, cold ischemia time was 459 min (217–886), which appears to be few hours shorter than most studies (32, 33), possibly having an impact on the incidence of DGF (34, 35). In a previous study, the tubular function slope (defined as the slope of the graft TAC using a linear fit between 30 and 60) was shown to be lower in patients with DGF, and predicted graft failure at 1 year (AUC 0.70) (36). Benjamins et al. described a similar parameter, the average upslope that was defined by the formula: (counts at 3 min—counts at 20 s)/160 s, which had high specificity and sensitivity to detect DGF one week after transplantation (37). Both these parameters are similar to our 3rd/2nd-minute activity ratio as they focus on the extraction/uptake phase occurring in the first minutes of RS. Our findings are thus consistent with others and confirm that this phase has the best predictive value for renal graft outcome. Our data suggest that RS may identify patients who will have adequate graft function at one year. Such knowledge could help selecting patients to undergo closer follow-up. It could be relevant for clinical decision making in several clinical settings (for instance hospital discharge, choice of immunosuppressive therapy). In addition, qualitative interpretation of RS is hindered by larger inter-observer variability. Although the use of a specific program to calculate these parameters may also have a learning curve (38), the 3rd/2nd-minute activity ratio seems to be a promising tool to improve inter-observer agreement as it is a simple ratio of activity counts with no further processing.

This study has several strengths. It is the first to evaluate the ability of RS to predict renal function specifically in patients undergoing DCD kidney transplantation. All RS were reviewed by two nuclear medicine specialists blinded to the outcome. Additional parameters, based on renal absolute counts, were calculated. Our findings appear robust, as they were confirmed by several sensitivity analyses. However, limitations need to be acknowledged. First, our cohort included only a small number of patients, thus with low power increasing the risk of type II errors and with potential confounding risk factors. In addition, this was a retrospective single-center study, which may affect statistical analysis. Finally, patients who did not have a RS after surgery were excluded, potentially excluding patients with superior renal function post-operatively. However, most DCD patients had a post-operative RS, regardless of their eGFR, and as part of our standardized clinical protocol.

## Conclusion

In conclusion, the RS parameter 3rd/2nd-minute activity ratio has a good specificity and sensitivity to predict DCD kidney graft function at one year post transplantation. The benefit of post-transplant RS should be further evaluated in larger prospective studies.

## Data Availability

The original contributions presented in the study are included in the article/**Supplementary Material**, further inquiries can be directed to the corresponding author/s.
