# Erratum: Role of post-transplant graft scintigraphy in kidney donation after circulatory death

**DOI:** 10.3389/frtra.2023.1171190

**Published:** 2023-03-17

**Authors:** Frontiers Production Office

**Affiliations:** Frontiers Media SA, Lausanne, Switzerland

**Keywords:** kidney transplantation, scintigraphy, outcome, DCD−donation after cardiac death, transplantation

An Erratum on Role of post-transplant graft scintigraphy in kidney donation after circulatory death By Belhoste M, Allenbach G, Agius T, Meier RPH, Venetz J, Corpataux J, Schneider A, Golshayan D, Prior JO, Déglise S, Nicod-Lalonde M and Longchamp A. (2022) Front. Transplant. 1:1065415. doi: 10.3389/frtra.2022.1065415

An omission to the funding section of the original article was made in error. The following sentence has been added: “Open access funding was provided by the University of Lausanne”.

The original version of this article has been updated.

