# Supplementary material for: Role of post-transplant graft scintigraphy in kidney donation after circulatory death
Source: Front Transplant. 2022 Dec 19;1:1065415. doi: 10.3389/frtra.2022.1065415 (PMC11235226; doi:10.3389/frtra.2022.1065415)
Supplement: Supplementary file 1 [file Datasheet1.pdf]

Supplementary Material

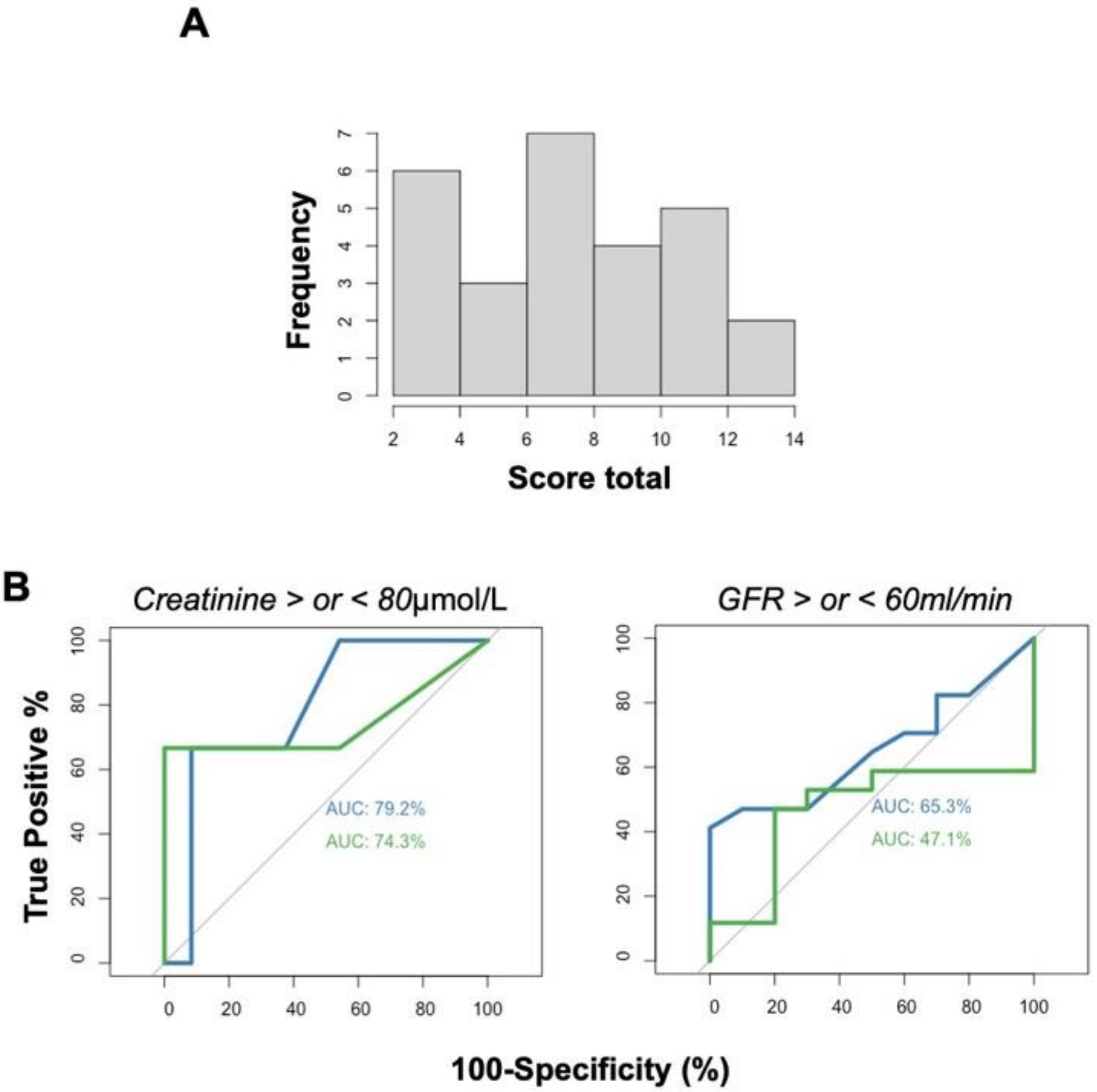

FIGURE S1. Evaluation of a new RS score. (A) Score distribution across the patients. (B) ROC curve illustrating the diagnostic ability of a new score to discriminate patients with eGFR above or below 80 µmol/L at 1 year. (C) ROC curve illustrating the diagnostic ability of a new score to discriminate patients with eGFR above or below 60 mL/min at 1 year.

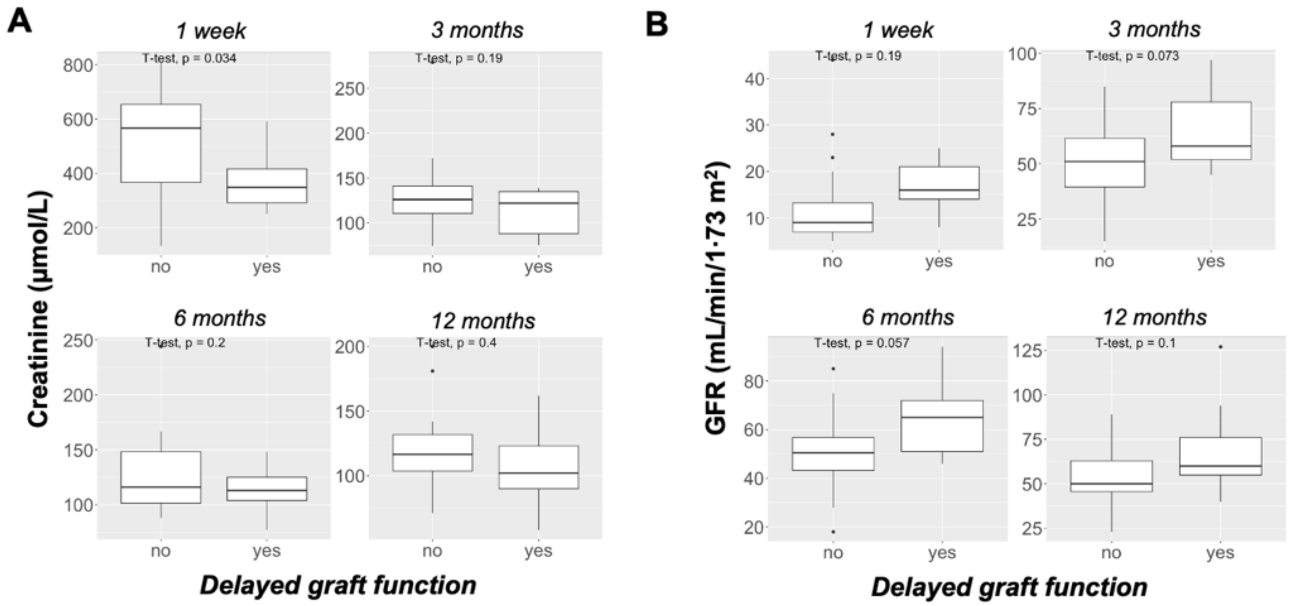

FIGURE S2. Serum creatinine level (A) and eGFR (B) at the indicated time as a function of delayed draft function.
